# Supplementary material for: Clinical outcomes of gastrointestinal bleeding management during anticoagulation therapy
Source: PLoS One. 2022 Jun 3;17(6):e0269262. doi: 10.1371/journal.pone.0269262 (PMC9165820; doi:10.1371/journal.pone.0269262)
Supplement: Attachment — Submitted filename: Response to reviewer.docx [file pone.0269262.s002.docx]

Response to reviewer’s comments

Reviewer #1:

1. Elaborate upon the Introduction and Discussion sections, discussing the rationale and the previous literature in greater depth. Keep the Discussion concise as well by ensuring that material in the Results section of the study are not frequently repeated in the Discussion section.

Response) Thank you for your review. We sincerely appreciate your high insight into our study. We changed and rearranged the introduction and discussion sections according to your recommendation. We made this section more concise and eliminated the repetition of results.

2. Ensure that units for all parameters are mentioned. For example, units of eGFR and admission duration should be mentioned in the tables.

Response) Thank you very much for the detailed point. As you pointed out, we added units for all parameters in the table

3. Discuss any bias that may have arisen and highlight future areas in which potential work/research may be done.

Response) In the Discussion, we added the following sentence at the High-risk thromboembolism subset section (line 280~284). “The result seems to be the effect that the physicians have established from the previous randomized clinical trial results that NOAC had less fatal bleeding than warfarin. It might be necessary to study the risk of further GI bleeding in patients who previously switched from warfarin to NOAC.” We added the following sentence in the Patients with valve replace surgery section (line 307~309). “With the case of previous ICH and GI bleeding, physicians’ treatment strategies should differ. Further studies on anticoagulant treatment strategies are needed for further clarification.”

4. We rearranged the discussion section; thus, the orders of references were changed. We add two references (#10 and #20).

Response) “10.	Barkun AN, Almadi M, Kuipers EJ, Laine L, Sung J, Tse F, et al. Management of Nonvariceal Upper Gastrointestinal Bleeding: Guideline Recommendations From the International Consensus Group. Ann Intern Med. 2019;171(11):805-22. Epub 2019/10/22. doi: 10.7326/M19-1795. PubMed PMID: 31634917; PubMed Central PMCID: PMCPMC7233308.

20.	Delate T, Meisinger SM, Witt DM, Jenkins D, Douketis JD, Clark NP. Bridge Therapy Outcomes in Patients With Mechanical Heart Valves. Clin Appl Thromb Hemost. 2017;23(8):1036-41. Epub 2016/09/23. doi: 10.1177/1076029616669786. PubMed PMID: 27655997.”
